# Increased risk of pneumonia in residents living near poultry farms: does the upper respiratory tract microbiota play a role?

**DOI:** 10.1186/s41479-017-0027-0

**Published:** 2017-02-25

**Authors:** Lidwien A. M. Smit, Gert Jan Boender, Wouter A. A. de Steenhuijsen Piters, Thomas J. Hagenaars, Elisabeth G. W. Huijskens, John W. A. Rossen, Marion Koopmans, Gonnie Nodelijk, Elisabeth A. M. Sanders, Joris Yzermans, Debby Bogaert, Dick Heederik

**Affiliations:** 1 Institute for Risk Assessment Sciences (IRAS), Division Environmental Epidemiology, Utrecht University, PO Box 80178, 3508 TD Utrecht, The Netherlands; 2 Central Veterinary Institute, Wageningen University and Research Centre, Lelystad, The Netherlands; 3 Department of Paediatric Immunology and Infectious Diseases, The Wilhelmina Children’s Hospital/University Medical Center Utrecht, Utrecht, The Netherlands; 4 Department of Medical Microbiology, Albert Schweitzer Hospital, Dordrecht, The Netherlands; 5 Laboratory of Medical Microbiology and Immunology, St. Elisabeth Hospital, Tilburg, The Netherlands; 6 Department of Medical Microbiology, University of Groningen, University Medical Center Groningen, Groningen, The Netherlands; 7 Department of Virology, Erasmus Medical Centre, Rotterdam, The Netherlands; 8 Current address: GD Animal Health, Deventer, The Netherlands; 9 National Institute for Public Health and the Environment, Bilthoven, The Netherlands; 10 NIVEL, Netherlands Institute for Health Services Research, Utrecht, The Netherlands

**Keywords:** Air pollution, Environment, Microbiome, Pneumonia

## Abstract

**Background:**

Air pollution has been shown to increase the susceptibility to community-acquired pneumonia (CAP). Previously, we observed an increased incidence of CAP in adults living within 1 km from poultry farms, potentially related to particulate matter and endotoxin emissions. We aim to confirm the increased risk of CAP near poultry farms by refined spatial analyses, and we hypothesize that the oropharyngeal microbiota composition in CAP patients may be associated with residential proximity to poultry farms.

**Methods:**

A spatial kernel model was used to analyze the association between proximity to poultry farms and CAP diagnosis, obtained from electronic medical records of 92,548 GP patients. The oropharyngeal microbiota composition was determined in 126 hospitalized CAP patients using 16S-rRNA-based sequencing, and analyzed in relation to residential proximity to poultry farms.

**Results:**

Kernel analysis confirmed a significantly increased risk of CAP when living near poultry farms, suggesting an excess risk up to 1.15 km, followed by a sharp decline. Overall, the oropharyngeal microbiota composition differed borderline significantly between patients living <1 km and ≥1 km from poultry farms (PERMANOVA *p* = 0.075). Results suggested a higher abundance of *Streptococcus pneumoniae* (mean relative abundance 34.9% vs. 22.5%, *p* = 0.058) in patients living near poultry farms, which was verified by unsupervised clustering analysis, showing overrepresentation of a *S. pneumoniae* cluster near poultry farms (*p* = 0.049).

**Conclusion:**

Living near poultry farms is associated with an 11% increased risk of CAP, possibly resulting from changes in the upper respiratory tract microbiota composition in susceptible individuals. The abundance of *S. pneumoniae* near farms needs to be replicated in larger, independent studies.

**Electronic supplementary material:**

The online version of this article (doi:10.1186/s41479-017-0027-0) contains supplementary material, which is available to authorized users.

## Background

Community-acquired pneumonia (CAP) is among the leading causes of morbidity and mortality in adults worldwide, and the burden is markedly higher among the oldest adults [1–3]. Environmental risk factors, such as active and passive smoking, household air pollution due to biomass fuel use, and outdoor air pollution, have been shown to increase the susceptibility to lower respiratory tract infections, including CAP [1, 4–7].

In a rural population in the south of The Netherlands, we previously studied environmental risk factors of CAP using electronic GP medical records of 70,142 adult patients seen during 2009 [8]. In this study, we observed an increased risk of CAP in adults living at a distance of 1 km or less from a poultry farm [8]. Although poultry farms can be a source of zoonotic pathogens such as avian influenza or *Chlamydia psittaci*, a study of zoonotic infections in hospitalized CAP patients in a nearby area revealed very few cases caused by avian pathogens (e.g. *C. psittaci*: 1.7%) [9].

Recently, scientists have sounded the alarm over the large contribution of agriculture to particulate matter (PM) air pollution [10, 11]. Poultry farms in particular are known to emit large quantities of air pollutants such as PM and endotoxins [12–15]. Exposure levels in stables have been shown to cause airway inflammation, respiratory symptoms and airway obstruction in farmers [16, 17]. Despite ambient pollutant concentrations being considerably lower than levels in stables, there is growing evidence of respiratory health effects in susceptible individuals living near livestock farms as well [18]. Exposure to PM air pollution may predispose these individuals to respiratory infections through chronic airway inflammation and subsequent host–immune responses [4, 7], which might be amplified by exposure to environmental endotoxin [19].

With the advent of new molecular microbial identification methods, it has been proposed that respiratory infections might emerge from the disruption of the otherwise balanced upper respiratory tract (URT) bacterial ecosystem or ‘microbiome’ [20]. Spread of overgrown pathogens originating from the URT to the lower respiratory tract could perturb the lung microbial ecosystem and lead to respiratory tract infections, including pneumonia [21]. Indeed, recent studies have shown that CAP in adults was associated with dysbiosis of the oropharyngeal microbiota, characterized by increased abundance of pathogens such as *S. pneumoniae*, and the absence of facultative anaerobic commensals [22]. We hypothesized that environmental exposure from poultry farms may contribute to a shift in the oropharyngeal microbiome, and thereby facilitate the development of lower respiratory infections.

In the present study, refined spatial analyses are used to confirm the increased risk of CAP near poultry farms. Furthermore, the oropharyngeal microbiota composition in CAP patients is analyzed in relation to residential proximity to poultry farms.

## Methods

### Study design

The study was carried out in the province of Noord-Brabant, The Netherlands, a region with a high density of intensive livestock farms. Two cross-sectional analyses were performed: (i) the association between poultry farms, home address and GP-registered pneumonia [8] was re-analyzed using a spatial kernel model; and (ii) the oropharyngeal microbiota composition of hospitalized CAP patients was analyzed in relation to residential proximity to poultry farms.

### Ethics

The study was carried out in accordance with Dutch legislation (the *Personal Data Protection Act*, and the *Medical Research Involving Human Subjects Act*) and with the Code of Conduct for Medical Research. All experimental protocols were approved by the Medical Ethical Committee of the University Medical Centre Utrecht (NL45307.041.13, GP-registered pneumonia; as part of the Farming and Neighbouring Residents’ Health Study), and by the Medical Ethical Committee of the St. Elisabeth Hospital, Tilburg (hospitalized CAP patients). Ethical aspects of the study have been described earlier [8, 23–25]. In short, medical information and address records of GPs’ patients were kept separated at all times by using a trusted third party. The need to obtain informed consent from individual patients was waived. Written informed consent was obtained from all hospitalized CAP patients.

### Spatial kernel analysis

Briefly, the data on GP-diagnosed pneumonia and spatial location of home address of 92,548 GP patients (70,142 adults [18–70 year] and 22,406 children [0–17 years]) during the year 2009 [8] was re-analyzed by estimating the parameters of a spatial kernel model from this data. In this model it is assumed that each poultry farm independently generates a probability for individuals of experiencing GP-diagnosed pneumonia within this year. This probability is modelled through a function dependent on the distance between farm and home address (i.e. the spatial kernel). The parameterization of the spatial kernel utilizes three parameters and offers sufficient flexibility for identifying any distance dependency from the data, from short-range to long-range patterns and from very sharp to more gradual dependencies on distance. In addition, a constant background probability is assumed to allow for quantification of the part of CAP incidence that is not associated with proximity to poultry farms. The background probability and the three parameters of the kernel are all estimated from the data by maximum likelihood (ML). The method is described previously [26, 27], and more details are provided in Additional file 1.

### Oropharyngeal microbiota composition

**Fig. 1:**
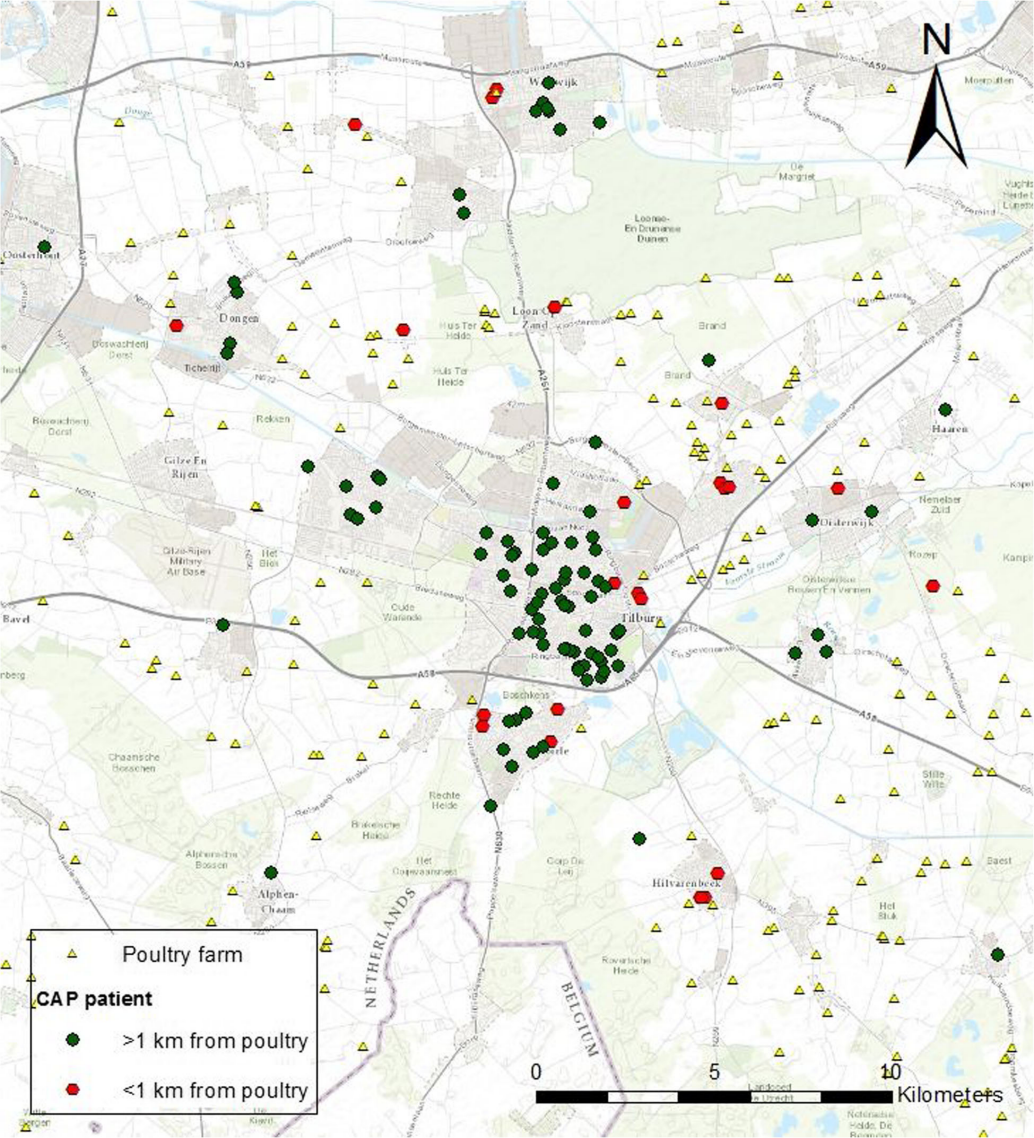
Geographic distribution of 126 adult CAP patients and poultry farms around Tilburg, The Netherlands

The microbiota composition of 126 patients hospitalized with CAP in the same region was analyzed, in relation to residential distance to poultry farms (Fig. 1). Characteristics, bacterial and viral etiology, and in-/exclusion criteria of the study population of hospitalized CAP patients have been described earlier [22, 25]. Oropharyngeal samples were taken before hospitalization at the emergency ward. Storage of samples, isolation of bacterial DNA, and 16S-rRNA-based sequencing of the V5–V7 region was performed as previously described [22]. Patients who received antibiotics during the two weeks before sampling, and patients with a positive qPCR result for *C. burnetii* were excluded from microbiota composition analysis. The present analysis was performed on 126 patients from whom microbiota data and address records were available. The presence of animal farms around the home address was assessed using a geographic information system (ARCGIS 9.3.1., Esri, Redlands, California, United States), as described earlier [9].

### Statistical analysis

Statistical analyses of oropharyngeal microbiota data were performed in R version 3.2.2. The study visualized the association between the vicinity of poultry farms and the overall bacterial community composition by non-metric multidimensional scaling (nMDS) of the Bray-Curtis dissimilarity metric and assessed its statistical significance by permutation analysis of variance [28]. Differentially abundant bacterial community members that might be related to changes in the overall microbiota composition were detected by Mann-Whitney U-tests, comparing patients living <1 km versus ≥1 km from poultry farms. The 1 km radius was chosen based on the original findings [8]. Sensitivity analyses were performed using a 1.15 km radius, based on the spatial kernel analysis results. The false discovery rate was controlled for by using the Benjamini-Hochberg technique [29]. Additionally, unsupervised hierarchical clustering was used to group patients based on similarity of microbial composition, which was visualized in a dendrogram [30]. The optimal number of clusters was based on clustering indices [22], and only clusters including >3 patients were considered for subsequent analyses. Fisher’s exact tests were exploited to compare the proportion of patients living <1 km from a poultry farm over all patients in the cluster of interest versus that proportion in the other clusters.

## Results

### Spatial kernel analysis

**Fig. 2:**
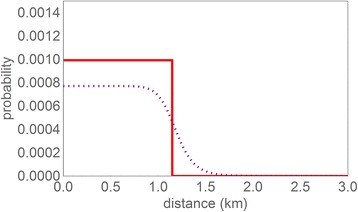
Spatial kernel estimated from morbidity data: probability for an individual of experiencing GP-diagnosed pneumonia in 2009 as attributed to an individual poultry farm, as a function of the distance from the individual’s residential location to that of the poultry farm. Red, full line: Maximum-Likelihood estimate (= fitted model). Grey, dotted line: Kernel for the lower confidence bound for the sharpness of the distance dependence and the corresponding profile-likelihood values for the other two parameters

The data was analyzed using spatial kernel models to characterize the association between living in the vicinity of poultry farms and CAP. In total, 993 patients (702/70,142 adults and 221/22,406 children) were diagnosed with CAP in 2009. Two alternative models were used: a distance-independent model with one estimable parameter (namely a constant per-individual probability of GP-reported pneumonia), and a model with a distance-dependent risk component (three additional estimable parameters) added to a constant background risk. Estimation of the distance-dependent risk component (spatial kernel) yields the results displayed in Fig. 2. The distance dependence of the fitted distance-dependent risk component (full line) is sharp, displaying a stepwise decline to zero at a distance of approximately 1.15 km. The excess CAP risk is approximately equal to 0.001 (per person year) per poultry farm within 1.15 km, which compares to a total background risk of 0.009 per person year (0.001/0.009 = ~11% increase in risk). Comparison with the distance-independent model shows that the distance-dependent component significantly contributes to the model fit (*p* = 0.0067, likelihood-ratio test, see Additional file 1). The sharp bound at 1.15 km of the distance-dependent risk model is largely maintained, even in the model including the 95% lower confidence bound value for the parameter determining the sharpness of the distance dependence (dashed line). For this lower confidence bound it was calculated (see Additional file 1) that 81% of the total CAP incidence attributed to proximity to individual poultry farms occurs within a 1.15 km distance from farms, and 95% within 1.35 km. To investigate potential age effects, kernel estimation was repeated for the partial datasets of 70,142 adults, and 22,406 children. No significant differences were observed between the parameter values estimated for these partial datasets, implying that both subpopulations show similar excess risks at close proximity from poultry farms (see Additional file 1).

### Oropharyngeal microbiota analysis

The oropharyngeal microbiota composition of 100 adult hospitalized CAP patients living <1 km and 26 patients living ≥1 km to one or more poultry farms was studied to explore the hypothesis that the association between CAP and living near poultry farms is mediated through a direct effect on microbial community composition, including pathogen colonization within the respiratory tract (Fig. 1).

Table 1 shows characteristics of the 26 CAP patients who lived within 1 km of one or more poultry farms, and 100 patients who lived at ≥1 km. Sex, age, smoking, chronic obstructive pulmonary disease (COPD), immunocompromized status, and pneumonia severity did not differ between the two groups (*p* > 0.25). All subjects were included in the same winter season, between November 2008 and February 2009. Bacterial and viral etiology also did not differ between the two groups (see Additional file 1: Table S3).

**Table 1:** Characteristics of 126 CAP patients, by the presence of a poultry farm within 1 km of the home address

	Poultry farm at ≥1 km	Poultry farm at <1 km	*p*-value^a^
n	100	26	
Male sex, n (%)	61 (61.0)	15 (57.7)	0.76
Age, mean ± SD, yrs	70.3 ± 15.3	66.5 ± 13.6	0.26
CAP at age ≥60 year, n (%)	80 (80.0)	19 (73.1)	0.44
Current smoking, n (%)	40 (40.0)	8 (30.8)	0.30
Month of admission, n (%)			
November 2008	17 (17.0)	6 (23.1)	0.71
December 2008	27 (27.0)	8 (30.8)	
January 2009	26 (26.0)	7 (26.9)	
February 2009	30 (30.0)	5 (19.2)	
Recent antibiotics usage^b^	0 (0.0)	0 (0.0)	NA
COPD, n (%)	40 (40.0)	10 (38.5)	0.89
ICS, n (%)	6 (6.0)	3 (11.5)	0.39
PSI, n (%)			
Mild	26 (26.0)	8 (30.7)	0.60
Moderate	45 (45.0)	13 (50.0)	
Severe	29 (29.0)	5 (19.2)	

^a^Chi-square test, Fisher’s exact test, or *t*-test. ^b^Use of antibiotics <2 weeks before admission

*COPD* chronic obstructive pulmonary disease, *NA* not available, *ICS* immunocompromized status, *PSI* pneumonia severity index

**Fig. 3:**
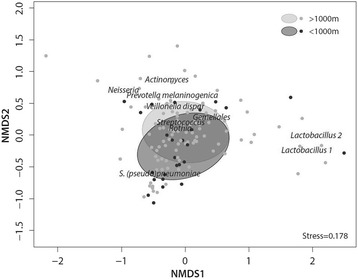
Non-metric multidimensional scaling (nMDS) plot of the oropharyngeal microbiota based on Bray-Curtis dissimilarity metric. Each data point depicts the oropharyngeal bacterial communities of one patient. Data points and population standard deviation of data points (ellipses) are colored based on vicinity to poultry farm (dark gray, <1 km; light gray, ≥1 km). The stress-value indicates that the multi-dimensional structure of the data is well captured by the nMDS visualization. The figure suggests an association between living close to a poultry farm and the abundance of *S. pneumoniae*, which was verified by both supervised and unsupervised learning methods

The association between living within a 1 km radius from a poultry farm and the overall oropharyngeal microbiota composition was borderline significant (Fig. 3, PERMANOVA *p* = 0.075). This overall difference in bacterial community composition was related to a higher abundance of *S. pneumoniae* (mean relative abundance 34.9% versus 22.5%, Mann-Whitney *U*-test *p* = 0.058, q = 0.541) and a lower abundance of *Lactobacillus* (1.4% versus 3.8%, *p* = 0.049, q = 0.541) in patients living at <1 km compared to ≥1 km from a poultry farm.

**Fig. 4:**
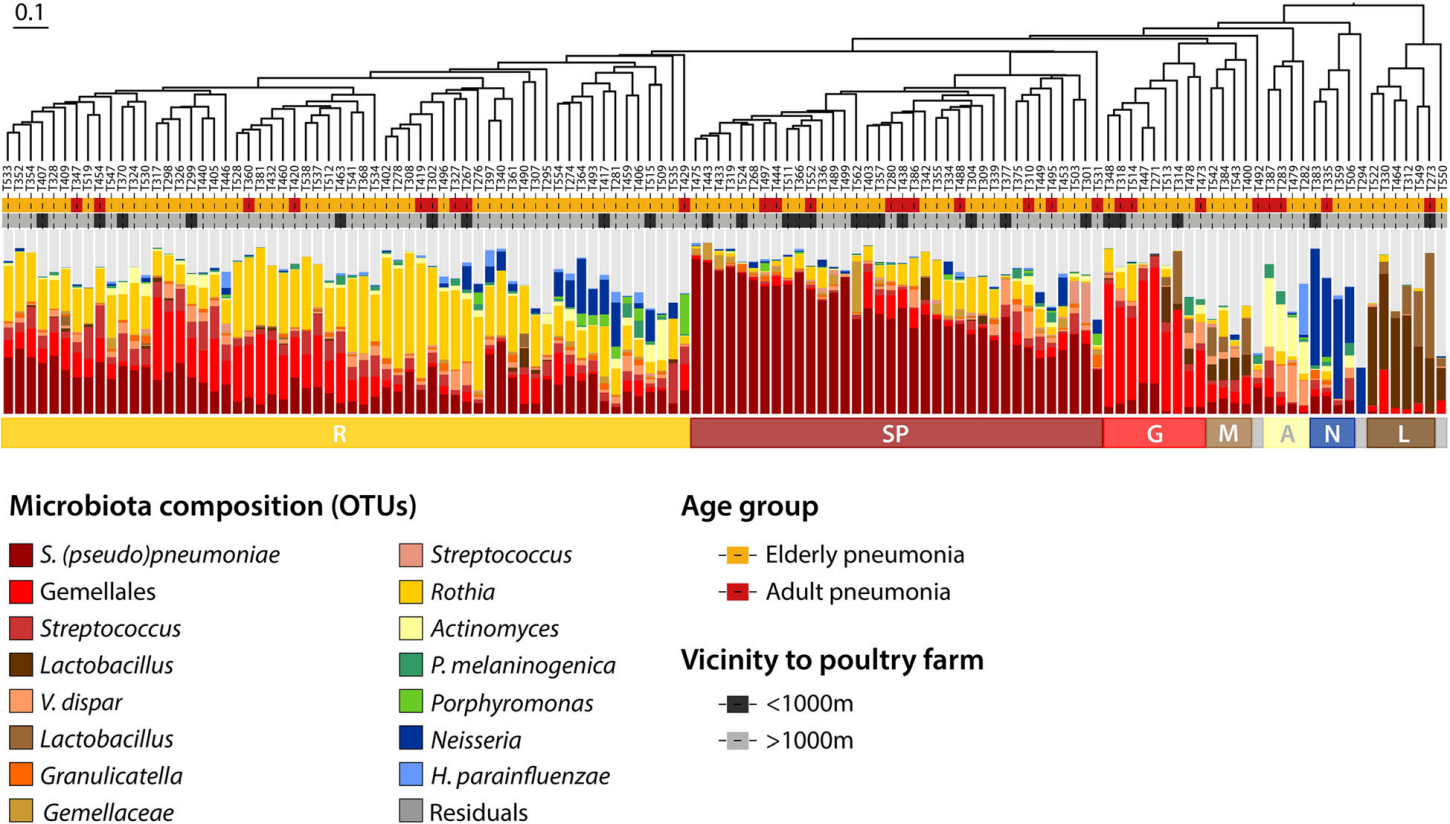
Hierarchical clustering of patients based on oropharyngeal microbiome composition. Patients were hierarchically clustered based on their oropharyngeal bacterial communities using the Bray-Curtis dissimilarity measure, which was visualized in the dendrogram. Adjacent to the branches of the dendrogram information on age (yellow; elderly, red; adults) and proximity to poultry farms (dark gray, <1 km; light gray, ≥1 km) is shown. In addition, the relative abundance of the 15 highest ranking operational taxonomic units (OTUs) is shown per patient in vertical stacked bar plots. The colored horizontal bars represent the three major clusters we discerned based on clustering indices which were enriched for *Rothia* (R), *S. pneumoniae* (SP) and *Gemellales* (G). Furthermore, four smaller clusters were observed, distinguished by predominance of *Actinomyces* (A), *Neisseria* (N) and *Lactobacillus* (L). In the fourth cluster no predominance for any OTU was observed (mixed; M)

These findings were verified using an unsupervised clustering approach, showing a total of seven microbiota clusters in our study cohort, six of which were characterized by either *Rothia*, *S. pneumoniae*, *Gemellales*, *Neisseria, Actinomyces* or *Lactobacillus* predominance. In addition, we identified one cluster without clear predominance of any species (Fig. 4). We observed an over-representation of individuals with the *S. pneumoniae* profile near poultry farms (12/36 versus 14/90 for the other clusters, Fisher’s exact *p* = 0.049). All remaining individual clusters, including *Rothia* (9/60 in close proximity versus 17/66 in the remaining clusters, *p* = 0.186) and *Gemellales* (3/9 in close proximity versus 23/117 in the remaining clusters, *p* = 0.390) showed no statistically significant associations with residential proximity to poultry farms. These findings suggest an association between living in close vicinity to poultry farms and enrichment for *S. pneumoniae* in particular.

We performed a sensitivity analysis, in which we compared the microbiota profiles of 98 CAP patients living <1.15 km and 28 patients living ≥1.15 km to one or more poultry farms, based on the spatial kernel analysis results. In patients who—compared to the primary analysis—were now considered “exposed” (living between 1.0 and 1.15 km from a poultry farm), the relative abundance of *S. pneumoniae* was below the 10th percentile, which attenuated the difference in the microbiota composition between the two groups (overall microbiota comparison: PERMANOVA *p* = 0.23, mean relative abundance of *S. pneumoniae*: Mann-Whitney *U*-test *p* = 0.14, over-representation of the *S. pneumoniae* cluster near poultry farms: Fisher’s exact *p* = 0.095).

## Discussion

In this study, we found that living near poultry farms is associated with an ~11% increase in risk of CAP for each poultry farm within a distance of 1.15 km. Furthermore, the results suggested an association between living close to a poultry farm and the abundance of *S. pneumoniae* in adult patients hospitalized with CAP. To the best of our knowledge, this study is the first to show an association between the residential environment and the respiratory microbiota composition in adults. Because of the lack of a healthy control group and the relatively small sample size, results should be interpreted with caution. The abundance of *S. pneumoniae* in relation to living in the vicinity of poultry/livestock farms needs to be reassessed in future studies.

Kernel analysis confirmed the excess risk of CAP in the vicinity of poultry farms, as identified earlier using regression analysis [8]. In addition, it indicated that the excess risk has a sharp bound at around 1.15 km. The advantage of the kernel analysis compared to earlier analyses is that instead of regressing CAP occurrence to distance-to-the-nearest-farm, it models the distance-dependent per-farm contribution to the risk of CAP. Thus, the kernel models can assess the incremental risk per every additional poultry farm as well as local accumulation of risk due to the presence of multiple farms in the vicinity of an individual’s home. In the earlier analyses, we considered an individual exposed when poultry farms were present within a 1 km radius of the home address. The distance dependence of the fitted kernel model is remarkably sharp, suggesting that the risk is strongly localized, possibly as a result of movement patterns around the home. This suggestion is supported by considering the kernel corresponding to the lower confidence bound of the parameter determining the sharpness of the distance dependence. Even for that kernel parameter set, we calculated that as much as 81% (on average) of the CAP incidence attributed to proximity to individual poultry farms occurs within a 1.15 km distance. This roughly corresponds to the earlier assumed 1 km radius around the home address. It should be realized that all risk estimates make use of the distance between a poultry farm and the home address as a proxy of exposure. Activity patterns lead to exposure misclassification and this may have modified the shape of the kernel function to some extent.

We hypothesized that the association between residential proximity to poultry farms and CAP might be mediated through an effect of emissions from poultry farms on microbiota composition in the URT. Our finding that the abundance of *S. pneumoniae* was increased in CAP patients living close to a poultry farm underlines our hypothesis that exposure to farm-related air pollutants may result in alterations of the oropharyngeal microbiota composition. An imbalanced URT microbiome may lead to decreased colonization resistance and reduced containment of commensals that may become pathogens (pathobionts) such as *S. pneumoniae*, consequently increasing the risk of respiratory tract infections [20].

We speculate that PM and endotoxin emissions from intensive agricultural operations are central to our findings, putatively by modulating innate immune responses resulting in an imbalanced respiratory microbiome, which is supported by animal and *in vitro* experiments [31, 32]. Alveolar macrophages remove inhaled PM by phagocytosis and release pro-inflammatory mediators, including reactive oxygen species. Their function is altered by particulate loading, which was shown to result in reduced phagocytosis of *S. pneumoniae* [33]. Furthermore, PM increases adhesion of *S. pneumoniae* to human airway epithelial cells, which is mediated by oxidative stress and upregulation of platelet-activating factor receptors (PAFR) [34, 35]. In aged rats, a single exposure to ambient PM reduced the ability to handle ongoing pneumococcal infections [36]. It was recently shown that the composition of the lung microbiota shifted towards endogenous opportunistic pathogens in a lipopolysaccharide (LPS) induced lung inflammation mouse model [32]. It has earlier been proposed that a perturbed respiratory microbiome may be involved in the association between cigarette smoking and respiratory tract infections. Both culture-based and culture-independent studies have shown that smoking can simultaneously deplete members of the normal commensal airway flora and enrich for potential pathogens [37]. Morris et al. [38] showed significant differences in the microbiome of the oral cavity of smokers compared with nonsmokers. We postulate similar mechanisms are responsible for the current observations. Smoking status (and other potential confounders such as COPD) were not associated with residential proximity to a poultry farm, thus it is unlikely that confounding has influenced the results.

In this study, bacterial community changes were observed on top of the previously described dysbiotic oropharyngeal microbiota composition in CAP patients compared to healthy adults, which was characterized by an increase of *S. pneumoniae*, *Rothia* and *Lactobacillus* [22]. Most strikingly, we showed an increased abundance of the most important human pathobiont causing pneumonia (i.e. *S. pneumoniae*) in oropharyngeal samples of patients living near poultry farms, which was a consistent finding across supervised and unsupervised learning methods. Our results are of great relevance for studies in individuals exposed to other sources of PM such as traffic and solid biomass fuel combustion, which are linked to acute respiratory infections as well [4, 5, 7], and may also be associated with an altered respiratory microbiota composition.

The observation of enhanced growth of a human pathobiont suggests an indirect effect of exposure to poultry farm emissions on pathogenesis of CAP. This is in contrast to a Finnish study [39], which observed that environmental biodiversity was associated with a higher generic diversity of skin gammaproteobacteria, presumably resulting from direct contact with environmental microbiota. Although there is some evidence that transmission of farm-related bacteria may take place through air—for example, in pig and veal calf farmers and their family members, nasal colonization by livestock-associated methicillin-resistant *Staphylococcus aureus* (LA-MRSA) occurs frequently [40]—we did not find evidence of zoonotic infections explaining the increased risk of CAP around poultry farms [9].

Around 40% of the CAP patients also had COPD. It has been demonstrated that COPD patients living near livestock farms are more likely to use inhaled corticosteroids and report respiratory symptoms than patients living further away from farms, suggesting an increased risk of exacerbations [18, 24]. Dickson et al. [41] proposed that exacerbations of COPD are occasions of respiratory dysbiosis: disorder and dysregulation of the microbial ecosystem of the respiratory tract, coupled with a dysregulated host immune response. A similar mechanism as proposed in the present study, hinging on farm-related air pollutants and dysbiosis of the respiratory tract, may play a role in individuals with COPD living near livestock farms. We intend to study a rural population of both COPD patients and control subjects in a larger-scale future study. Including control subjects will help to interpret results, because the microbiota changes within patient groups may be a cause or a consequence of disease, or merely coinciding with disease status [22].

Indeed, a limitation of our preliminary microbiota study is that we did not include healthy controls from the same study area. However, hospitalized patients living near a poultry farm and those living further away from poultry farms did not differ with regard to potential confounders such as age, smoking, COPD, or pneumonia severity. Residual confounding due to demographic differences between the two groups (e.g. household size), contact with children or other risk factors for pneumonia or pneumococcal colonization cannot be entirely dismissed. Because of the relatively small sample size, the results are sensitive to the effect of outliers. In addition, there may be false positive results due to multiple comparisons. This should therefore be regarded as a hypothesis-generating study, and results need to be confirmed in larger, preferably longitudinal, studies.

## Conclusions

Kernel analysis demonstrated an excess risk of GP-diagnosed pneumonia in areas of approximately 1.15 km around poultry farms. Oropharyngeal microbiota of patients with CAP living within 1 km of a poultry farm showed an increased abundance of *S. pneumoniae*. Exposure to air pollutants such as PM and endotoxin may contribute to dysbiosis of URT microbiota in susceptible individuals, which may consequently result in respiratory infections. These preliminary observations need to be replicated in larger, independent studies.

## Additional file

Additional file 1: Table S1. Kernel parameter and background probability estimates obtained using ML estimation, with 95% confidence bounds based on the likelihood-ratio test. **Table S2.**: Comparison between the distance-dependent en distance-independent model. **Table S3.** Viral and bacterial etiology of CAP, by the presence of a poultry farm within 1 km of the home address. (DOCX 122 kb)
