# Supplementary material for: Increased risk of pneumonia in residents living near poultry farms: does the upper respiratory tract microbiota play a role?
Source: Pneumonia (Nathan). 2017 Feb 25;9:3. doi: 10.1186/s41479-017-0027-0 (PMC5471663; doi:10.1186/s41479-017-0027-0)
Supplement: Additional file 1: Table S1. — Kernel parameter and background probability estimates obtained using ML estimation, with 95% confidence bounds based on the likelihood-ratio test. Table S2.: Comparison between the distance-dependent en distance-independent model. Table S3. Viral and bacterial etiology of CAP, by the presence of a poultry farm within 1 km of the home address. (DOCX 122 kb) [file 41479_2017_27_MOESM1_ESM.docx]

**ADDITIONAL FILE 1**

**SUPPLEMENTARY MATERIAL**

**Increased pneumonia risk near poultry farms: does the respiratory microbiota play a role?**

Lidwien A. M. Smit, Gert Jan Boender, Wouter A. A. de Steenhuijsen Piters, Thomas J. Hagenaars, Elisabeth G. W. Huijskens, John W. A. Rossen, Marion Koopmans, Gonnie Nodelijk, Elisabeth A. M. Sanders, Joris Yzermans, Debby Bogaert, Dick Heederik

**Kernel analysis: Methods**

Data on GP-diagnosed pneumonia over the year 2009 of 92,548 individuals living in a region with high farm density in the South East of The Netherlands were available as a subset of the morbidity data of a study described in [S1]. Location coordinates of all poultry farms in the study area were obtained from the provincial database of mandatory environmental licenses for keeping livestock in 2009. Coordinates of the GP patients’ residential locations were obtained by geocoding the residential addresses.

The kernel is a probability, independently generated by each individual poultry farm, for individuals of experiencing GP-diagnosed pneumonia within the year 2009. This probability $p$ is assumed to only depend on the distance between poultry farm and residential location: $p=p(r_{ij})$, where $r_{ij}$ is the straight-line distance between poultry farm $i$ and the residential location of individual $j$. We write $p\left(r\right)=1-\exp\left(-\lambda\left(r\right)\right)$ in which $\lambda\left(r\right)$ is a hazard function. The kernel hazard function is parameterized as follows

| . |  |
| --- | --- |

Here the parameters $\lambda_{0},\alpha$ and $r_{0}$ determine the scale and the shape of the distance-dependent component of $\lambda\left(r\right)$. This parameterization is adopted from the best-fitting hazard model identified in an analysis of between-flock transmission of Highly Pathogenic Avian Influenza [S2]. The parameter $\lambda_{0}$ determines the magnitude of the distance-dependent component at short distances $r\to0$, the parameter $r_{0}$governs the distance range around which the distance-dependent component is declining to zero, and the parameter $\alpha$ determines the sharpness of that decline, with values of $\alpha$ above 3 corresponding to sharp decline and values below 2 yielding a substantial long-distance tail. We introduce a distance-independent background pneumonia probability $P_{b}$, allowing for quantification of the component of pneumonia incidence that is not associated with proximity to poultry farms. Correspondingly, we define a distance-independent background hazard $\lambda_{b}=\ln\left(1-P_{b}\right)$.

We compare a full model including both distance-dependent and background hazards with a model consisting of only a distance-independent hazard.

The background hazard $\lambda_{b}$ and the kernel parameters $\lambda_{0},\alpha,r_{0}$ are estimated using Maximum-Likelihood (ML). We calculate 95% confidence intervals for the parameters using the likelihood-ratio test. The likelihood function is defined as

| $L=\prod_{j\in\text{Λ}^{\text{NP}}} p_{\text{esc},j}\prod_{m\in\text{Λ}^{\text{P}}} \left(1-p_{\text{esc},m}\right)$. |  |
| --- | --- |

Here, $p_{esc,j}$and $p_{esc,m}$ denote the total probability of an individual $j$and individual $m$, respectively, to stay free of GP-diagnosed pneumonia, $\Lambda^{\text{NP}}$ denotes the set of all patients in the study population without GP-diagnosed pneumonia in 2009, and $\Lambda^{\text{P}}$ denotes the set of all patients in the study population with GP-diagnosed pneumonia in 2009. From the assumed independence of the probabilities generated by each one of the poultry farms it follows that total probability $p_{esc,j}$ of an individual $j$ to stay free of GP-diagnosed pneumonia is given by $p_{esc,j}=\left(1-P_{b}\right)\prod_{i} p_{esc,ji}$, where the index $i$ runs over all flocks in the study area, $1-P_{b}$ is the probability of an individual escaping from GP-diagnosed pneumonia caused by the background hazard, and $p_{esc,ji}=1-p\left(r_{ij}\right)$ is the probability of an individual $j$ escaping from GP-diagnosed pneumonia caused by farm $i$. Using the relationship $p\left(r\right)=1-\exp\left(-\lambda\left(r\right)\right)$, we may write $p_{esc,ji}=\exp\left(-\lambda\left(r_{ij}\right)\right)$, and thus the likelihood may be written in terms of $\lambda\left(r\right)$ as follows:

$$L=\prod_{j\in\text{Λ}^{\text{NP}}} \exp\left(-\lambda_{b}-\sum_{i} \lambda\left(r_{ij}\right)\right)\prod_{m\in\text{Λ}^{\text{P}}} \left(1-\exp\left(-\lambda_{b}-\sum_{i} \lambda\left(r_{im}\right)\right)\right).$$

**Kernel analysis: Parameter estimates**

The Maximum-Likelihood (ML) estimates and univariate confidence bounds for the kernel parameters are presented in Table S1. These results underlie the graphs shown in Figure 2 in the main text.

As already stated in the main text, the analysis was carried out three times: For the full dataset of patients, for the partial dataset of adult patients (18-70yrs), and for the partial dataset of children (0-17yrs). No significant differences were observed between the parameter values estimated for these three datasets. Remarkably, the parameter $\alpha$, which determines how sharply the spatial correlation is declining around the distance range determined by $r_{0}$, is estimated to be extremely large ($\infty$). This estimate corresponds to an almost stepwise decline to zero of the distance dependent component of the kernel, occurring at the distance $r=r_{0}.$ Even the lower confidence bound estimated for $\alpha$ from the full dataset corresponds to a rather sharp decline, as depicted by the dotted line in Figure 2 in the main text. The comparison between the distance-dependent en distance-independent model is given in Table S2, and shows that the distance-dependence is significant (p<0.05).

**Table S1.** Kernel parameter and background probability estimates obtained using ML estimation, with 95% confidence bounds based on the likelihood-ratio test.

|  | $\lambda_{0}$(×10^-3^) | $\alpha$ | $r_{0}$ (km) | $P_{b}$ (×10^-3^) |
| --- | --- | --- | --- | --- |
| All | 0.99 (0.47–1.53) | $\infty$ (12.3,$\infty$) | 1.15 (1.11,1.33) | 8.99 (8.44,9.80) |
| Adults | 0.84 (0.2–3.14) | $\infty$ (2.8,$\infty$) | 1.15 (0.37,1.46) | 9.20 (8.36,10.12) |
| Children | 1.30 (0.37–2.52) | $\infty$ (4.8,$\infty$) | 1.23 (0.87,1.66) | 8.27 (6.90,9.93) |

**Table S2.** Comparison between the distance-dependent en distance-independent model.

| **Model comparison** | **Parameter difference** | **AIC difference** | **p value of likelihood-ratio test** |
| --- | --- | --- | --- |
| Background + distance-dependent effect of poultry farms *versus* Background only | 3 | 8.22 | 0.0067 |

**Kernel analysis: Calculation of the percentage of the total pneumonia incidence attributed to proximity to individual poultry farms occurring within a given distance from poultry farms**

In order to investigate how sharply an excess pneumonia risk is localized within the vicinity of a poultry farm, we use the model to calculate which percentage (or proportion) of the total pneumonia incidence attributed to proximity to poultry farms is attributed to farms within a given distance from the patient’s home location. In detail, the proportion $f\left(r\leq d\right)$ within a distance $d$ is calculated as:

$f\left(r\leq d\right)=\frac{\sum_{i} \sum_{\begin{matrix} m\in\text{Λ}^{\text{P}} \\ r_{im}\leq d \end{matrix}} \lambda\left(r_{im}\right)}{\sum_{i} \sum_{m\in\text{Λ}^{\text{P}}} \lambda\left(r_{im}\right)}$.

Here the summation with index $i$ is over all poultry farms in the dataset, and $\Lambda^{\text{P}}$ denotes the set of all patients in the study population with GP-diagnosed pneumonia in 2009.

**Table S3.** Viral and bacterial etiology of CAP, by the presence of a poultry farm within 1 km of the home address.

|  | Poultry farm at ≥1 km | Poultry farm at <1 km | p-value^a^ |
| --- | --- | --- | --- |
| n | 100 | 26 |  |
| Etiology (%) |  |  | 0.599 |
| Not identified | 35 (35.0) | 7 (26.9) |  |
| Bacterial | 34 (34.0) | 8 (30.8) |  |
| Viral | 13 (13.0) | 6 (23.1) |  |
| Mixed | 18 (18.0) | 5 (19.2) |  |
| *Streptococcus pneumoniae* (%) | 42 (42.0) | 10 (38.5) | 0.918 |
| *Legionella pneumophila* (%) | 1 (1.0) | 0 (0.0) | 1.000 |
| *Coxiella burnetii* (%) | 1 (1.0) | 0 (0.0) | 1.000 |
| *Staphylococcus aureus* (%) | 2 (2.0) | 1 (3.8) | 1.000 |
| *Pseudomonas aeruginosa* (%) | 1 (1.0) | 1 (3.8) | 0.878 |
| *Escherichia coli* (%) | 1 (1.0) | 1 (3.8) | 0.878 |
| *Mycoplasma pneumoniae* (%) | 0 (0.0) | 0 (0.0) | NA |
| *Chlamydia psittaci* (%) | 1 (1.0) | 0 (0.0) | 1.000 |
| *Chlamydia pneumoniae* (%) | 1 (1.0) | 0 (0.0) | 1.000 |
| Adenovirus (%) | 0 (0.0) | 0 (0.0) | NA |
| Human bocavirus (%) | 2 (2.0) | 0 (0.0) | 1.000 |
| KI polyomavirus (%) | 1 (1.0) | 0 (0.0) | 1.000 |
| WU polyomavirus (%) | 1 (1.0) | 0 (0.0) | 1.000 |
| Human metapneumovirus (%) | 2 (2.0) | 0 (0.0) | 1.000 |
| Human rhinovirus (%) | 3 (3.0) | 1 (3.8) | 1.000 |
| Human coronavirus (OC43) (%) | 3 (3.0) | 2 (7.7) | 0.597 |
| Human coronavirus (NL63) (%) | 3 (3.0) | 1 (3.8) | 1.000 |
| Human coronavirus (HKU) (%) | 0 (0.0) | 0 (0.0) | NA |
| Human coronavirus (229E) (%) | 0 (0.0) | 0 (0.0) | NA |
| Human parainfluenzavirus (1) (%) | 4 (4.0) | 1 (3.8) | 1.000 |
| Human parainfluenzavirus (2) (%) | 0 (0.0) | 0 (0.0) | NA |
| Human parainfluenzavirus (3) (%) | 0 (0.0) | 0 (0.0) | NA |
| Human parainfluenzavirus (4) (%) | 0 (0.0) | 0 (0.0) | NA |
| Influenza A virus (%) | 15 (15.0) | 3 (12.0) | 0.949 |
| Influenza B virus (%) | 3 (3.0) | 1 (4.0) | 1.000 |
| Respiratory syncytial virus (%) | 3 (3.0) | 3 (12.0) | 0.174 |

^a^Chi-square test or Fisher’s exact test; NA: Not applicable. Detection methods are described in detail by Huijskens et al. [S3]. Data are presented as n (%).

**References**

[S1]. Smit LA, van der Sman-de Beer F, Opstal-van Winden AW, Hooiveld M, Beekhuizen J, Wouters IM, Yzermans J, Heederik D. Q Fever and pneumonia in an area with a high livestock density: a large population-based study. PLoS One 2012; 7: e38843.

[S2]. Boender GJ, Hagenaars TJ, Bouma A, Nodelijk G, Elbers ARW, De Jong MCM, Van Boven M. Risk maps for the spread of highly pathogenic avian influenza in poultry. PLoS Comput Biol 2007; 3: e71.

[S3]. Huijskens EG, van Erkel AJ, Palmen FM, Buiting AG, Kluytmans JA, Rossen JW. Viral and bacterial aetiology of community-acquired pneumonia in adults. Influenza Other Respir Viruses. 2013;7:567-573.
